# Arabidopsis CAPRICE (MYB) and GLABRA3 (bHLH) Control Tomato (*Solanum lycopersicum*) Anthocyanin Biosynthesis

**DOI:** 10.1371/journal.pone.0109093

**Published:** 2014-09-30

**Authors:** Takuji Wada, Asuka Kunihiro, Rumi Tominaga-Wada

**Affiliations:** 1 Graduate School of Biosphere Sciences, Hiroshima University, Higashi-Hiroshima, Hiroshima, Japan; 2 Faculty of Applied Biological Science, Hiroshima University, Higashi-Hiroshima, Hiroshima, Japan; NARO Institute of Fruit Tree Science, Japan

## Abstract

In *Arabidopsis thaliana* the MYB transcription factor CAPRICE (CPC) and the bHLH transcription factor GLABRA3 (GL3) are central regulators of root-hair differentiation and trichome initiation. By transforming the orthologous tomato genes *SlTRY* (*CPC*) and *SlGL3* (*GL3*) into *Arabidopsis*, we demonstrated that these genes influence epidermal cell differentiation in Arabidopsis, suggesting that tomato and *Arabidopsis* partially use similar transcription factors for epidermal cell differentiation. CPC and GL3 are also known to be involved in anthocyanin biosynthesis. After transformation into tomato, *35S::CPC* inhibited anthocyanin accumulation, whereas *GL3::GL3* enhanced anthocyanin accumulation. Real-time reverse transcription PCR analyses showed that the expression of anthocyanin biosynthetic genes including *Phe-ammonia lyase* (*PAL*), the flavonoid pathway genes *chalcone synthase* (*CHS*), *dihydroflavonol reductase* (*DFR*), and *anthocyanidin synthase* (*ANS*) were repressed in *35S::CPC* tomato. In contrast, the expression levels of *PAL*, *CHS*, *DFR*, and *ANS* were significantly higher in *GL3::GL3* tomato compared with control plants. These results suggest that *CPC* and *GL3* also influence anthocyanin pigment synthesis in tomato.

## Introduction

Anthocyanins are important chemical compound of polyphenolic pigments derived from the phenylpropanoid biosynthetic pathway. Anthocyanins belong to the group of flavonoids, of which they are noticeable in the wide range of chemical structures [1]. Anthocyanins provide appealing color to leaves, flowers, fruits and seeds in plants. In addition to this obvious feature, they have other essential functions. Anthocyanin synthesis was induced by the stressful occasions, such as low temperature or strong irradiation of the sunlight, against which they protect the plant as scavengers for radical species or a light-screen [2]. Anthocyanins are produced through several enzymatic step [3]. The enzymes which are involved in anthocyanin synthesis are fully analyzed by both biochemical and genetic approaches.

Thus, it is important to identify the regulatory factors governing this enzymatic steps. In *Arabidopsis*, anthocyanin biosynthesis is regulated by the TTG1-bHLH-MYB protein complex [4]–[10]. In *Arabidopsis*, overexpressions of *PAP1/MYB75*, *PAP2/MYB90*, *MYB113* and *MYB114*, which are R2R3-type MYB transcription factors, accelerate the anthocyanin accumulations in *Arabidopsis* [10], [11]. Two homologous bHLH proteins, GLABRA3 (GL3) and ENHANCER OF GLABRA3 (EGL3) enhance anthocyanin biosynthesis together with PAP1 and PAP2 [7]. In contrast, *CAPRICE* (*CPC*), one of R3-type MYB genes, compete with the binding of PAP1/2 to GL3/EGL3 and disrupt the TTG1-GL3/EGL3-PAP1/2 protein complex, thus inhibiting the activity of anthocyanin biosynthesis [12].

*CPC* has been initially identified as a key regulator of root-hair differentiation in *Arabidopsis thaliana* [13]. *Arabidopsis* has six additional *CPC*-like MYB genes in its genome, including *TRYPTICHON* (*TRY*), *ENHANCER OF TRY AND CPC1* (*ETC1*), *ENHANCER OF TRY AND CPC2* (*ETC2*), *ENHANCER OF TRY AND CPC3/CPC-LIKE MYB3* (*ETC3/CPL3*), *TRICHOMELESS1* (*TCL1*), and *TRICHOMELESS2/CPC-LIKE MYB4* (*TCL2/CPL4*) [14]–[22]. These *CPC*-like MYB family genes cooperatively regulate Arabidopsis epidermal cell differentiation including root-hair and trichome formation [14]–[23].

*GL3* is also important for root-hair and trichome differentiation in *Arabidopsis* [24]. The gene products of *GL3*, *EGL3* [25], *WEREWOLF* (*WER*), which encodes an R2R3 type MYB protein [26] and *TRANSPARENT TESTA GLABRA1* (*TTG1*), which encodes a WD-40 protein [27] form a transcriptional complex [7], [24], [28]. This protein complex, including the WER, GL3/EGL3 and TTG1 proteins, controls transcription of the *GLABRA2* (*GL2*) gene [29]. The *GL2* gene encodes a homeodomain leucine zipper protein and is thought to act farthest downstream in the Arabidopsis root-hair and trichome differentiation regulatory pathway [13], [26], [27], [30], [31]. CPC moves form non-hair cells to hair cells where it disrupts TTG1-GL3/EGL3-WER transcriptional complex by competing the binding of WER [32].

In the previous study, we identified Arabidopsis *CPC* and *GL3* homologous genes from tomato and named them *Solanum lycopersicum TRYPTICHON* (*SlTRY*) and *Solanum lycopersicum GLABRA3* (*SlGL3*), respectively [33]. The *SlTRY*-encoded protein was most closely related to TRY among the CPC-like MYBs [33]. Transformants expressing the tomato *TRY* homologous gene (*SlTRY*) in Arabidopsis had a greater number of root-hairs and no trichomes, a phenotype similar to that seen in over-expressors of *CPC*-like MYB genes. On the other hand, transformants expressing the tomato *GL3* homologous gene (*SlGL3*) in Arabidopsis had no obvious *GL3*-like phenotypes related to non-hair and trichome cell differentiation [33]. We concluded that tomato and Arabidopsis use similar transcription factors for root-hair and trichome cell differentiation and that the *SlTRY*-like R3 MYB may be a key common regulator of plant root-hair and trichome development [33]. In prior work, we also analyzed the anthocyanin content of *SlTRY* and *SlGL3* transgenic Arabidopsis [34]. We showed that anthocyanin accumulation was repressed in the *CPC::SlTRY* and *GL3::SlGL3* transgenic Arabidopsis plants, suggesting that the tomato genes of *SlTRY* and *SlGL3* are involved in anthocyanin biosynthesis [34].

In this study, we have expressed the Arabidopsis *CPC* and *GL3* genes in tomato to show the effect of these genes on tomato anthocyanin biosynthesis, indicating that *GL3* is a positive regulator for anthocyanin biosynthesis, but *CPC* is a negative regulator.

## Materials and Methods

### Plant materials and growth conditions

Tomato, *Solanum lycopersicum* L. cv. Micro-Tom, was used. Seeds were surface-sterilized with 10% commercial bleach including a detergent (Kitchen Haiter, Kao, Tokyo, Japan), for 20 min and then rinsed with sterilized water three times for 5 min each and sown on 1.5% agar plates containing 0.5× MS medium [35]. Seeded plates were held at 4°C for 2 d and then incubated at 25°C under constant white light (50–100 µmol m^−2^ s^−1^) for 7 days to produce seedlings for RNA extraction. Some 7-day-old seedlings were transplanted into soil and grown in a photoperiod of 16 h light (50–100 µmol m^−2^ s^−1^) at 25°C for an additional week to produce mature plant tissues for anthocyanin extraction.

### Transgenic plants

Gene constructs of *35S::CPC* [13] and *GL3::GL3* [36] were introduced into tomato (Micro-Tom) according to a highly efficient transformation protocol for Micro-Tom [37]. *Agrobacterium tumefaciens* C58C1 was grown for 24 h at 28°C. Cotyledon explants were sectioned, dipped in the bacterial suspension to allow adsorption, and transferred to callus induction medium containing 100 mg L^−1^ kanamycin, 1.5 mg L^−1^ zeatin and 375 mg L^−1^ Augmentin (GlaxoSmithKline, Uxbridge, UK) [37]. Transgenic shoots were selected and rooted on a medium containing 50 mg/L kanamycin.

Homozygous transgenic lines were selected based on kanamycin resistance. We obtained ten and four T2 transgenic tomato lines and selected eight and three homozygous lines of *35S::CPC* and *GL3::GL3*, respectively. The presence of *35S::CPC* and *GL3::GL3* in the transgenic plants was confirmed by PCR using *CPC* or *GL3* forward and reverse primers (Table 1) (Figure S1). Only those plants with the expected PCR products (*CPC* and *GL3*) were used in the analyses.

**Table 1:** Primer sequences used in this study.

Primer Name	Sequence (5′ to 3′)
CPC-F	5′-GGATGTATAAACTCGTTGGCGACAG-3′
CPC-R	5′-GCCGTGTTTCATAAGCCAATATCTC-3′
GL3-F	5′-GATAACCATCGCAGGACTAAGC-3′
GL3-R	5′-CCCACTCAAGACTACTCACTTCTG-3′
PAL-F	5′-ATTGGGAAATGGCTGCTGATT-3′
PAL-R	5′-TCAACATTTGCAATGGATGCA-3′
CHS-F	5′-TGGTCACCGTGGAGGAGTATC-3′
CHS-R	5′-GATCGTAGCTGGACCCTCTGC-3′
DFR-F	5′-CAAGGCAGAGGGAAGATTCATTTG-3′
DFR-R	5′-GCACCATCTTAGCCACATCGTA-3′
ANS-F	5′-GAACTAGCACTTGGCGTCGAA-3′
ANS-R	5′-TTGCAAGCCAGGCACCATA-3′
LeActin-F	5′-TGTCCCTATTTACGAGGGTTATGC-3′
LeActin-R	5′-CAGTTAAATCACGACCAGCAAGAT-3′

### Real-time reverse transcription PCR analysis

The sequences of all primers used in this study are listed in Table 1. Total RNA from tomato tissues was extracted with MagDEA RNA 100 (GC) (PSS, Chiba, Japan) using a Magtration System 12 GC (PSS, Chiba, Japan). To remove contaminating genomic DNA, RNA samples were treated with RNase-free DNase I (Ambion, Austin, TX, USA) according to the Magtraction System protocol. Plant tissue (100 mg) was homogenized using a TissueLyser II (Qiagen, Valencia, CA, USA) with 100 µL of RLT buffer (Qiagen, Valencia, CA, USA). Sample supernatants were applied to the instrument, and RNA was eluted with 50 mL of sterile distilled water.

First-strand cDNA was synthesized from 1 µg total RNA in a 20 µL reaction mixture using the Prime Script RT Master Mix (Perfect Real Time) (Takara, Tokyo, Japan). Real-time PCR was performed using a Chromo4 Real-Time IQ5 PCR Detection System (Bio-Rad, Hercules, CA, USA) with SYBR Premix Ex Taq II (Takara, Tokyo, Japan). PCR amplification employed a 30 s denaturing step at 95°C, followed by 5 s at 95°C and 30 s at 60°C with 40 cycles for *CPC*, *GL3*, *PAL*, *CHS*, *DFR*, *ANS* and *LeActin*. Real-time PCR was used to analyze the mRNA expression level of each transcript encoding *CPC*, *GL3*, *PAL*, *CHS*, *DFR* and *ANS* in transgenic tomato. The relative expression of each transcript was calculated by the ΔΔCT method [38]. The expression levels of *CPC*, *GL3*, *PAL*, *CHS*, *DFR* and *ANS* were estimated after being normalized to the endogenous control gene *LeActin* (TC116322) [39]. The primers were: *CPC-F* and *CPC-R* for *CPC*; *GL3-F* and *GL3-R* for *GL3*; *PAL-F* and *PAL-R* for *PAL*; *CHS-F* and *CHS-R* for *CHS*; *DFR-F* and *DFR-R* for *DFR*; *ANS-F* and *ANS-R* for *ANS*; *LeActin-F* and *LeActin-R* for *LeActin* [39]–[41].

### Extraction and analysis of anthocyanins

Anthocyanin levels were measured according to previously reported protocols [42], [43]. Control and transgenic plants were grown together in a growth chamber as described above. Anthocyanins were extracted from cotyledons of 7-day-old seedlings, leaves and stems of three-week-old plants, and fresh weights were determined. Total plant pigments were extracted overnight in 0.3 mL acidic methanol (1% (v/v) HCl). After the addition of 0.2 mL water and an equal volume of chloroform, anthocyanins were separated from the chlorophylls by partitioning into the aqueous methanol phase, and the absorption was measured at 530–657 nm in a spectrometer (GENios, TECAN). Anthocyanin levels were then normalized to the total fresh weight of tissue used in each sample.

### Light microscopy

To observe anthocyanin pigment localization in hypocotyls of the control, *35S::CPC* and *GL3::GL3* transgenic plants, we prepared hand-cut sections from 3-week-old plants and observed them by light microscopy using a Zeiss (Jena, Germany) Axio Imager. Z1 microscope.

## Results

### Anthocyanin pigmentation of the *35S::CPC* and *GL3::GL3* transgenic plants

To establish whether Arabidopsis CPC and GL3 transcription factors function in tomato, we introduced these genes into one of tomato cultivars (*Solanum lycopersicum* L. cv. Micro-Tom). Previously, we showed that *35S::CPC* transgenic Arabidopsis plants have an unusually large number of root-hairs and no leaf trichomes [13]. Thus, we chose to introduce the *35S::CPC* construct into tomato in this experiment. In contrast, the root-hair number of *35S::GL3* transgenic Arabidopsis plants is not significantly different from the wild-type [25], suggesting that the 35S promoter is not suitable for *GL3* gene overexpression. The expression of *GL3* should be precisely controlled by the *GL3* promoter [31]. Therefore, we decided to use the *GL3::GL3* construct (a genome fragment of *GL3* driven by the *GL3* promoter) for transformation of tomato in this study [36].

**Figure 1:**
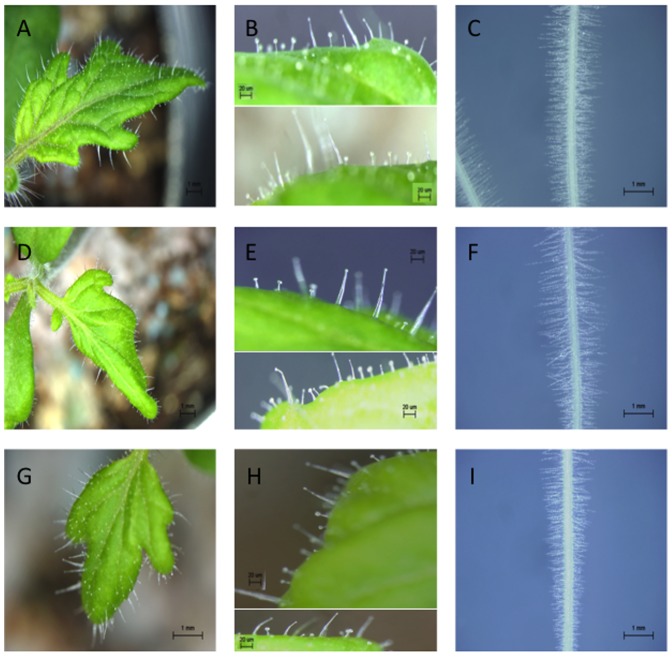
Leaf and root epidermal phenotypes of *35S::CPC* and *GL3::GL3* transgenic tomato plants. (A) The first true leaf from the two-week old control plant. (B) Close-up view of the adaxial side of the leaf shown in A. (C) Five-day-old seedling roots of control plants. (D) The first true leaf from the two-week old *35S::CPC* plant. (E) Close-up view of the adaxial side of the leaf shown in E. (F) Five-day-old seedling roots of *35S::CPC* plants. (G) The first true leaf from the two-week old *GL3::GL3*plant. (H) Close-up view of the adaxial side of the leaf shown in G. (I) Five-day-old seedling roots of *GL3::GL3* plants. Scale bars: 1 mm in A, C, D, F, G and I; 20 µm in B, E and H.

**Figure 2:**
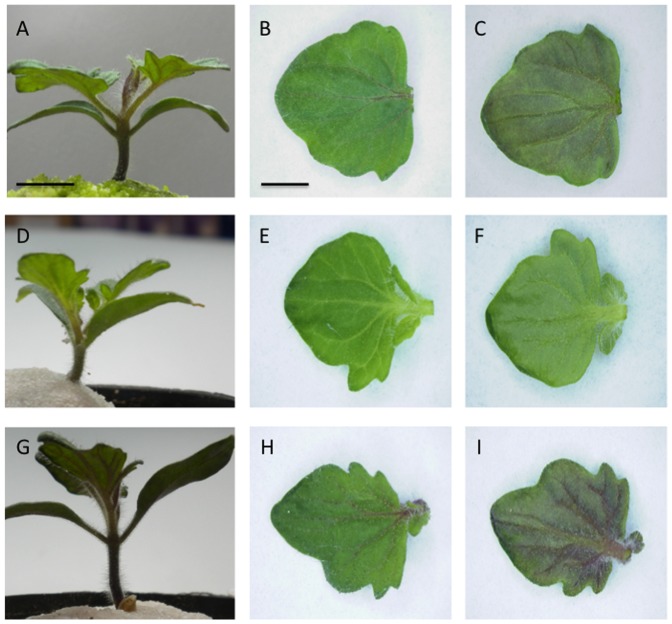
Phenotypes of *35S::CPC* and *GL3::GL3* transgenic tomato plants. (A) Two-week old control plant. (B) Adaxial side of the first true leaf from the plant shown in A. (C) Abaxal side of the first true leaf from the plant shown in A. (D) Two-week-old *35S::CPC* transgenic plant. (E) Adaxial side of the first true leaf from the plant shown in D. (F) Abaxal side of the first true leaf from the plant shown in D. (G) Two-week-old *GL3::GL3* transgenic plant. (H) Adaxial side of the first true leaf from the plant shown in G. (I) Abaxal side of the first true leaf from the plant shown in G. Scale bars: 1 cm in A for A, D and G; 5 mm in B for B, C, E, F, H and I.

The *35S::CPC* and *GL3::GL3* transgenic tomato plants were phenotypically similar to the control plants (Figure 1; Figure S1). We did not detect any remarkable differences between *35S::CPC* or *GL3::GL3* transgenic tomato plants and the control tomato plant in root-hair and trichome phenotypes (Figure 1; Figure S1). On the other hand, we observed qualitatively less and more reddish-purple coloration in the stems and leaves of *35S::CPC* and *GL3::GL3* plants, respectively (Figure 2A, 2D and 2G). The first true leaves of two-week-old *35S::CPC* transgenic plants had clearly lower amounts of anthocyanin pigmentation on the adaxial and abaxial sides of the leaves compared with that of the control plants (Figure 2B, 2C, 2E and 2F). Control plant leaves accumulated reddish-purple anthocyanin mainly in the leaf veins on the adaxial side and nearly the entire surface of the abaxial side of the leaves (Figure 2B, 2C). Leaf veins of the *35S::CPC* plants were pale green and no anthocyanin accumulation was observed on either side of the leaves (Figure 2E and 2F). On the other hand, leaves of the *GL3::GL3* plants accumulated greater amounts of reddish-purple anthocyanin in the leaf veins compared with the control plants (Figure 2H, 2I).

**Figure 3:**
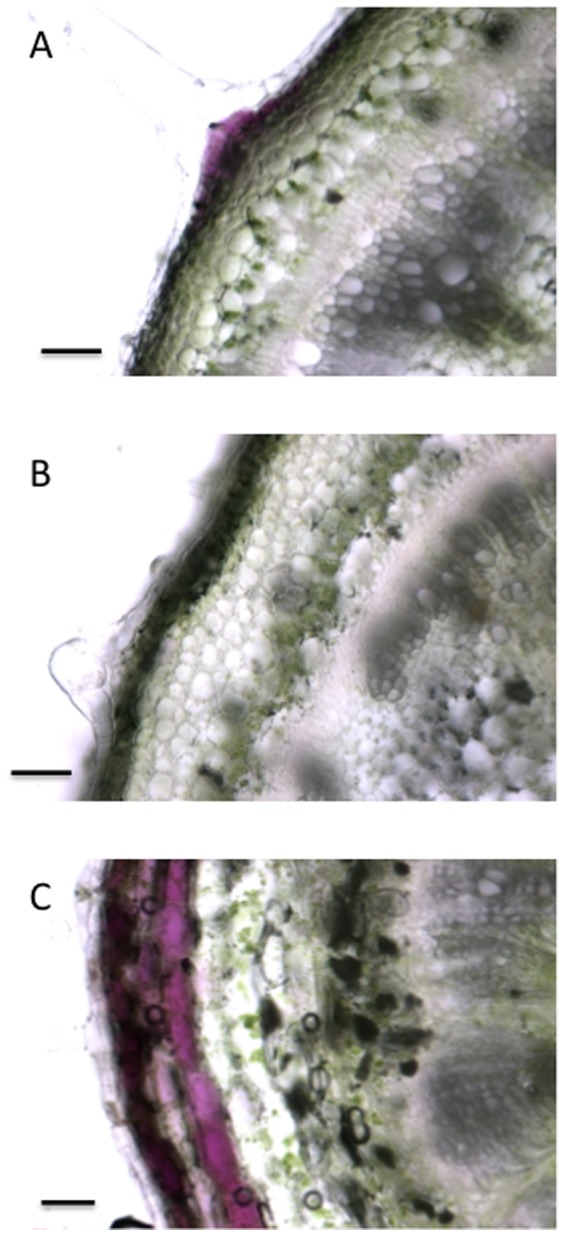
Stem phenotypes of *35S::CPC* and *GL3::GL3* transgenic tomato plants. (A) Transverse section of a hypocotyl of a two-week-old control plant. (B) Transverse section of a hypocotyl of a two-week-old *35S::CPC* transgenic plant. (C) Transverse section of a hypocotyl of a two-week-old *GL3::GL3* transgenic plant. Scale bars: 100 µm.

To determine the tissue distribution of anthocyanin pigments in the *35S::CPC* and *GL3::GL3* transgenic tomato plants, we examined hand-cut sections prepared from stem samples with a light microscope as shown in Figure 2A, 2D and 2G. In hypocotyls of two-week-old control tomato seedlings, anthocyanin pigments were observed in a few cells, as was previously reported in tomato hypocotyls (Figure 3A) [44]. Anthocyanins did not accumulate in the hypocotyls of young *35S::CPC* tomato seedlings (Figure 3B). In the hypocotyls of *GL3::GL3* seedlings, anthocyanin pigments were present in two to three layers of an epidermal cell and subepidermal cells (Figure 3C). These results suggest that *CPC* expression did not induce any remarkable changes in root-hair and trichome formation but reduced anthocyanin accumulation in transgenic tomato. *GL3* also did not affect the epidermal phenotype but induced anthocyanin accumulation in transgenic tomato.

### Analysis of anthocyanin levels in the cotyledons, leaves and stems of transgenic plants

**Figure 4:**
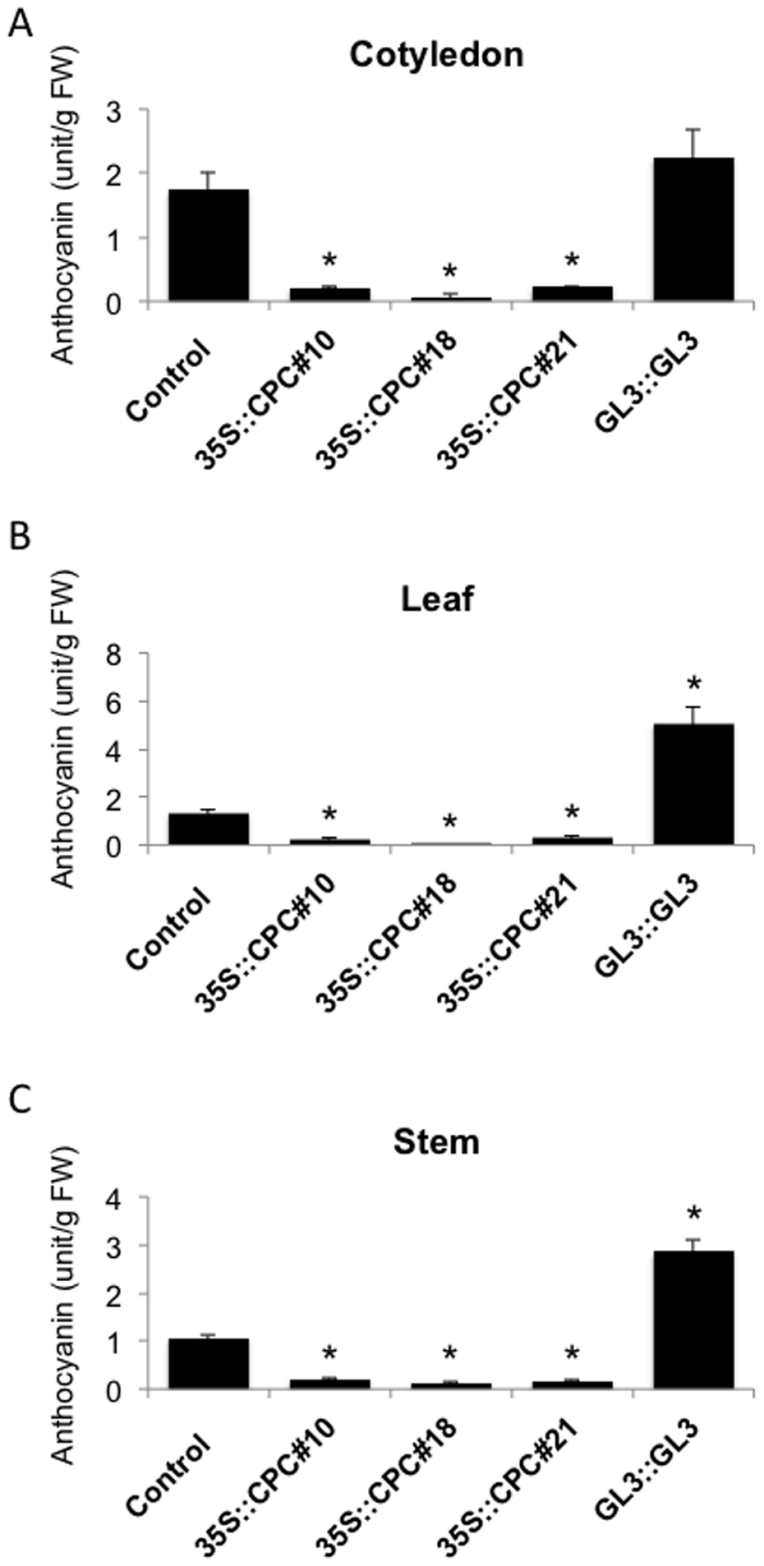
Anthocyanin content in control, *35S::CPC* and *GL3::GL3* transgenic tomato plants. (A) The anthocyanin content of cotyledons from control, *35S::CPC#10*, *35S::CPC#18*, *35S::CPC#21* and *GL3::GL3* plants are shown. (B) The anthocyanin content in leaves of control, *35S::CPC#10*, *35S::CPC#18*, *35S::CPC#21* and *GL3::GL3* plants are shown. (C) The anthocyanin content in stems of control, *35S::CPC#10*, *35S::CPC#18*, *35S::CPC#21* and *GL3::GL3* plants are shown. Error bars indicate the standard deviations. Bars marked with asterisks indicate a significant difference between the control and the *35S::CPC* or the *GL3::GL3* transgenic lines by Student's *t*-test (P<0.050).

We examined the effects of *CPC* and *GL3* on anthocyanin accumulation in the different tissues. Expression levels of the introduced *CPC* gene were checked by PCR, and we selected three lines (*35S::CPC*#10, *35S::CPC* #18 and *35S::CPC* #21) among eight transgenic lines for analysis (Figure S2A). Expression levels of the introduced *GL3* gene were also checked by PCR. Among three *GL3::GL3* transgenic lines, only one line, *GL3::GL3#12*, showed stable expression of *GL3*. Therefore, we used the *GL3::GL3#12* line for further analyses (Figure S2B). To compare the levels of anthocyanin accumulation in *35S::CPC* and *GL3::GL3* with those in control tomato, the anthocyanin content in extracts of two-week-old seedlings was determined (Figure 4). Compared with the control tomato cotyledons, all three lines of *35S::CPC* transgenic tomato cotyledons had significantly reduced levels of anthocyanin (Figure 4A). On the other hand, cotyledons of *GL3::GL3* accumulated higher levels of anthocyanin compared with that of the control plants (Figure 4A). Consistent with the observations shown in Figure 2, very low levels of anthocyanin accumulation were observed in leaves of all three *35S::CPC* lines (Figure 4B). Compared with control tomato leaves, significantly larger amounts of anthocyanin were measured in *GL3::GL3* leaves (Figure 4B). Consistent with the observations shown in Figure 2 and 3, anthocyanin accumulation was also significantly reduced in the stems of all three *35S::CPC* lines and increased in *GL3::GL3* stems compared with those in the control plants (Figure 4C). We confirmed that introduction of the *CPC* gene under the control of the 35S promoter significantly inhibited anthocyanin accumulation in cotyledons, leaves and stems of tomato as observed in Arabidopsis [12]. Introduction of the *GL3* gene under the control of the *GL3* promoter significantly increased anthocyanin accumulation also in mature leaves and stems of tomato as observed in Arabidopsis [45].

### Effect of *CPC* and *GL3* on the expression of anthocyanin pathway genes

**Figure 5:**
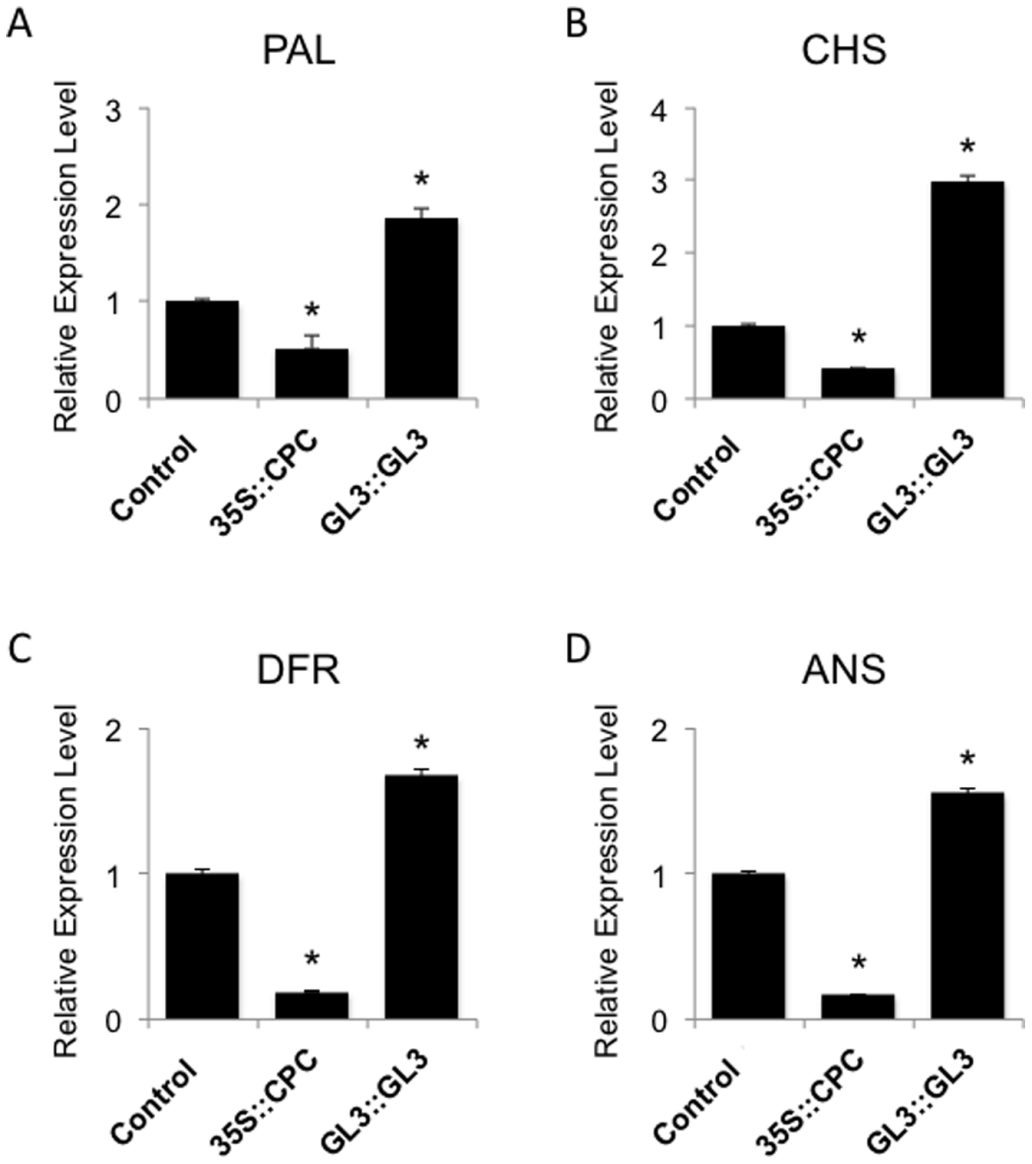
Expression analysis of genes associated with the anthocyanin biosynthetic pathway in tomato leaves. Enzyme names of the anthocyanin biosynthetic pathway are abbreviated as follows: phenyl alanine ammonia-lyase (PAL), chalcone synthase (CHS), dihydroflavonol 4-reductase (DFR), leucoanthocyanidin dioxygenase (ANS). (A) Real-time reverse transcription PCR analysis of *PAL* gene expression in *35S::CPC* and *GL3::GL3* transgenic tomato leaves. (B) Real-time reverse transcription PCR analysis of *CHS* gene expression in *35S::CPC* and *GL3::GL3* transgenic tomato leaves. (C) Real-time reverse transcription PCR analysis of *DFR* gene expression in *35S::CPC* and *GL3::GL3* transgenic tomato leaves. (D) Real-time reverse transcription analysis of *ANS* gene expression in *35S::CPC* and *GL3::GL3* transgenic tomato leaves. Total RNA was isolated from the indicated leaves from two-week-old tomato plants. Expression levels of *PAL*, *CHS*, *DFR and ANS3* in each sample relative to those in the control plants are shown. The experiments were repeated three times. Error bars indicate the standard error. Bars marked with asterisks indicate a significant difference between control and indicated transgenic plants by Student's *t*-test (P<0.050).

To characterize more fully the involvement of the introduced CPC and GL3 transcription factors on the regulation of anthocyanin biosynthesis in tomato, we examined the expression levels of genes that encode anthocyanin biosynthetic enzymes. The effects of *CPC* and *GL3* on the expression of anthocyanin biosynthesis genes were examined by real-time RT-PCR, as described in the Materials and Methods section. First and second true-leaf samples of representative *35S::CPC*, *GL3::GL3* and control plants, harvested from two-week-old seedlings, were homogenized, and total RNA was isolated from each tissue sample. Anthocyanins are synthesized through the flavonoid biosynthetic pathway [46]. Therefore, expression levels of tomato genes for *Phe-ammonia lyase* (*PAL*), the flavonoid pathway genes *chalcone synthase* (*CHS*), *dihydroflavonol reductase* (*DFR*), and *anthocyanidin synthase* (*ANS*) were determined and expressed relative to the *LeActin* gene, a tomato gene that encodes an actin protein [39]. Consistent with the reduced anthocyanin accumulation in *35S::CPC* transgenic tomato (Figure 4B), *PAL*, *CHS*, *DFR* and *ANS* expression levels were significantly lower in *35S::CPC* transgenic tomato compared with the control plants (Figure 5). In contrast, consistent with the large amount of anthocyanin accumulation in *GL3::GL3* transgene tomato (Figure 4B), *PAL*, *CHS*, *DFR* and *ANS* expression levels were significantly higher in *GL3::GL3* transgenic tomato compared with control plants (Figure 5). These results suggest that Arabidopsis *CPC* and *GL3* can regulate gene expression of the anthocyanin biosynthetic pathway in tomato and affect the anthocyanin accumulation.

## Discussion

In this study, we introduced the Arabidopsis *CPC* and *GL3* genes into tomato under the control of the *35S* promoter and the *GL3* promoter, respectively. Overexpression of *CPC* is known to induce root-hair cell differentiation and inhibits trichome formation in Arabidopsis [13]. Overexpression of *GL3* is known to reduce root-hair cell differentiation and induce trichome formation in Arabidopsis [25], [31]. However, overexpression of *CPC* and *GL3* in tomato did not result in visible differences in the root-hair and trichome phenotypes (Figure 1; Figure S1). The reasons for the differences in CPC and GL3 function between tomato and Arabidopsis may arise from fundamental differences in the way epidermal organs develop in the two plants. Root epidermal development in vascular plants is classified into three types [47]. Tomato root epidermal development belongs to type 1, in which root-hairs can be produced from any root epidermal cell [48]. Conversely, Arabidopsis root epidermal development belongs to type 3 in which root-hair cell files and non-hair cell files are organized in the root epidermis [47]. Regulation of root-hair cell and non-hair cell fate determination by the TTG1-GL3/EGL3-WER complex and CPC might be specific for Arabidopsis but not for tomato.

Trichome phenotypes are also different between Arabidopsis and tomato. Arabidopsis trichomes are normally large single cells with three branches [49], whereas tomato trichomes are chemically and morphologically divergent [50]–[52]. Tomato trichomes are classified into seven types, including glandular (types I, IV, VI and VII), and non-glandular (types II, III and V) trichomes [51], [53]. The participation of many regulatory genes might be necessary to form tomato trichomes. Hence, it is likely difficult to change tomato trichome phenotypes by *CPC* or *GL3* overexpression only. Tomato might need other transcriptional factors to change the morphology of the epidermal cell.

In a previous study, we isolated *SlTRY* and *SlGL3* from tomato as orthologous genes of the Arabidopsis *CPC* and *GL3*, respectively [33]. The full length SlTRY protein shares 50% amino acid identity with CPC [33]. Phylogenic analysis suggested that *SlTRY* and *CPC* originated from a single common ancestor [33]. *SlTRY* was shown to function quite similarly to the Arabidopsis *CPC*, including in the formation of ectopic root-hairs, in the induction of a no-trichome phenotype and in its action as a repressor of anthocyanin accumulation in Arabidopsis [34]. In summary, SlTRY functions in a similar way as CPC for the epidermal cell differentiation and the anthocyanin accumulation in Arabidopsis. On the other hand, there was no obvious effect on trichome or non-hair cell differentiation in the Arabidopsis *GL3::SlGL3* transformants [33]. Rather, anthocyanin accumulation was reduced in the *GL3::SlGL3* transgenic Arabidopsis compared with the wild-type [34]. In contrast, GL3 functions as a positive regulator for the anthocyanin accumulation in Arabidopsis [7]. The difference of the sequence between GL3 and SlGL3 might contribute to the opposite functions although they share 45% amino acid identity at the entire region [33]. Taken together, the functions of SlGL3 are completely different from those of GL3.

In this study, we demonstrated that Arabidopsis *CPC* and *GL3* genes regulate anthocyanin biosynthesis in tomato. We made *35S::CPC* transgenic tomatoes that accumulated significantly less anthocyanin in comparison with the control plants (Figure 4). In contrast, anthocyanin accumulation in *GL3::GL3* transgenic tomato was greater than the control plants (Figure 4). *CPC* and *GL3* are known to regulate anthocyanin biosynthesis in Arabidopsis [12], [54]. Our study suggests that the regulatory system for anthocyanin biosynthesis by *CPC* and *GL3* is maintained in both Arabidopsis and tomato.

Genes encoding enzymes of the anthocyanin biosynthetic pathway are divided into two groups: early biosynthetic genes including *PAL* and *CHS*, and late biosynthetic genes including *DFR* and *ANS*. The two groups have independent activation mechanisms in dicotyledonous species [55], [56]. Whereas *PAL* and *CHS* are involved in the synthesis of precursors and flavonoids, *DFR* and *ANS* are more specific for the synthesis of anthocyanins. Analysis of the biosynthetic pathway genes in tomato showed that genes of both groups were regulated by *CPC* and *GL3*. Expression levels of *PAL*, *CHS DFR* and *ANS* were significantly lower in *35S::CPC* transgenic tomato compared with the control plants (Figure 5). In contrast, expression levels of *PAL*, *CHS DFR* and *ANS* were significantly higher in *GL3::GL3* transgenic tomato compared with the control plants (Figure 5). *GL3* and *CPC* were strong up- and down-regulators of the entire anthocyanin biosynthesis pathway in tomato, respectively (Figure 5), which reflect the results form Arabidopsis [7], [12]. These results suggest the presence of a TTG1-TT8/GL3-PAP1/2 like protein complex that specifically regulates anthocyanin biosynthesis in tomato [45], [57]–[59].

Many studies contributed to the elucidation of the anthocyanin biosynthetic pathway using Arabidopsis [10], [60]–[64]. As a result, the molecular genetics of the regulatory system for anthocyanin biosynthesis has greatly progressed [1], [46], [65]–[67]. In Arabidopsis, the regulatory protein complex, which includes WD40, bHLH and MYB transcription factors, regulates anthocyanin biosynthesis [10], [58], [68], [69]. WD40 is encoded by *TTG1*, bHLHs are encoded by *TT8*, *GL3* and *EGL3*, and MYBs are encoded by *PAP1*, *PAP2*, *MYB113* and *MYB114* [65]. In addition to the WD40-bHLH-MYB complex, CPC, a single repeat R3-MYB, is a negative regulator of anthocyanin biosynthesis in Arabidopsis [12]. *MYBL2*, another R3-MYB gene, functions as a negative regulator of anthocyanin biosynthesis in Arabidopsis seedlings [70], [71]. Our study suggests the existence of a WD40-bHLH-MYB complex that regulates anthocyanin biosynthesis in tomato. CPC may disrupt this putative WD40-bHLH-MYB protein complex, thus inhibiting the activity of downstream anthocyanin biosynthetic genes in tomato. In Arabidopsis, there are a total of seven *CPC* family R3-type MYB genes, including *CPC*, *TRY*, *ETC1 ETC2*, *ETC3/CPL3*, *TCL1* and *TCL2/CPL4* [14]–[22]. In contrast, only *SlTRY* was identified as a putative tomato ortholog of *CPC* so far [33]. Although the total number of tomato *CPC* orthologous gene(s) is still unknown, fewer genes are expected than are present in the Arabidopsis genome. The small number of R3-type MYB gene(s) in tomato might reflect their specific functions in anthocyanin biosynthesis. Because *SlGL3* did not induce anthocyanin accumulation in Arabidopsis [34], *SlGL3* probably does not participate in the putative WD40-bHLH-MYB protein complex in tomato as is the case in Arabidopsis. A model for regulating anthocyanin biosynthesis in tomato by WD40-bHLH-MYB will be forthcoming with further analyses.

## Supporting Information

Figure S1 **Root and leaf epidermal phenotypes of** ***35S::CPC*** **and** ***GL3::GL3*** **transgenic tomato plants.** Five-day-old seedlings (left panels) and two-week-old plants (right panels) from control (top), *35S::CPC* (middle) and *GL3::GL3* (bottom) transgenic plants.
(TIFF)

Figure S2 ***CPC*** **or** ***GL3*** **expression in the transgenic tomato plants.** (A) Real-time reverse transcription PCR analyses of the *CPC* gene in eight *35S*::*CPC* (#6, #10, #15, #18, #20, #21, #24 and #26) transgenic plants. Expression levels of *CPC* in each line are reported relative to that of transgenic line #10. (B) Real-time reverse transcription PCR analyses of the *GL3* gene in three *GL3*::*GL3* (#4, #12 and #22) transgenic plants. Expression levels of *GL3* in each line are reported relative to that of transgenic line #4. Expression levels were normalized to *Act2* expression. The experiment was repeated three times. Error bars indicate the standard deviations.
(TIFF)
