# Supplementary figures and images for: Arabidopsis CAPRICE (MYB) and GLABRA3 (bHLH) Control Tomato (Solanum lycopersicum) Anthocyanin Biosynthesis
Source: PLoS One. 2014 Sep 30;9(9):e109093. doi: 10.1371/journal.pone.0109093 (PMC4182634; doi:10.1371/journal.pone.0109093)

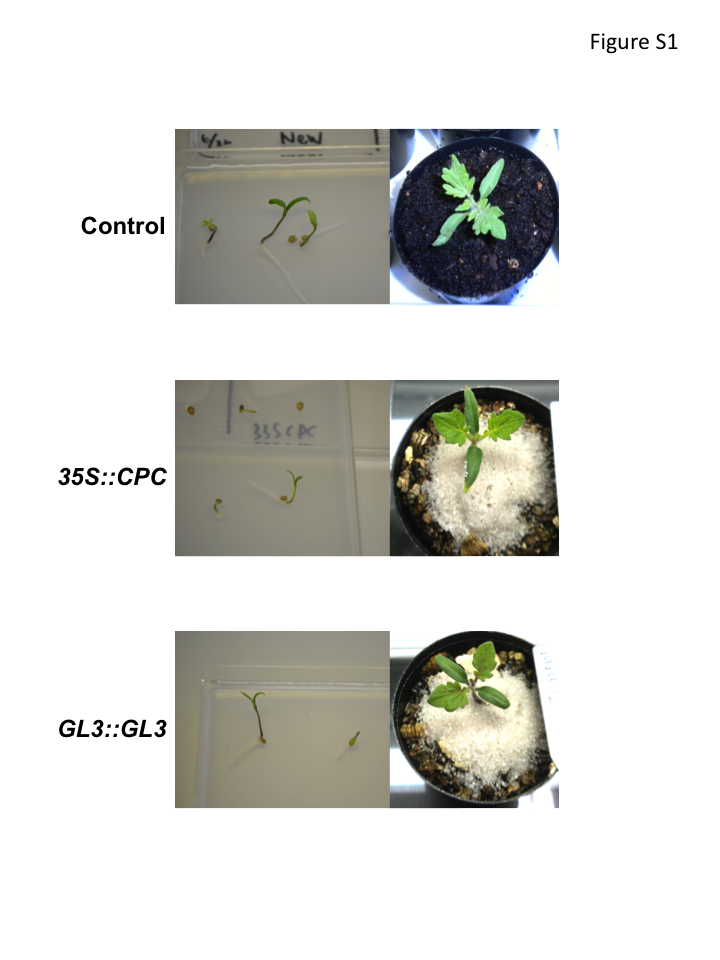

Supplement: Figure S1 — Root and leaf epidermal phenotypes of 35S::CPC and GL3::GL3 transgenic tomato plants. Five-day-old seedlings (left panels) and two-week-old plants (right panels) from control (top), 35S::CPC (middle) and GL3::GL3 (bottom) transgenic plants. (TIFF) [file pone.0109093.s001.tiff]
